# Oligoclonal Band Straightening Based on Optimized Hierarchical Warping for Multiple Sclerosis Diagnosis

**DOI:** 10.3390/s22030724

**Published:** 2022-01-18

**Authors:** Farah Haddad, Samuel Boudet, Laurent Peyrodie, Nicolas Vandenbroucke, Julien Poupart, Patrick Hautecoeur, Vincent Chieux, Gérard Forzy

**Affiliations:** 1 Biomedical Signal Processing Unit (UTSB), Lille Catholic University, F-59000 Lille, France; laurent.peyrodie@junia.com; 2 Faculty of Medicine and Midwifery (FMM), Lille Catholic Institute (ICL), F-59800 Lille, France; patrick.hautecoeur@univ-catholille.fr (P.H.); gerard.forzy@cegetel.net (G.F.); 3 Laboratoire d’Informatique Signal et Image de la Côte d’Opale (LISIC), Université du Littoral Côte d’Opale (ULCO), F-62228 Calais, France; nicolas.vandenbroucke@univ-littoral.fr; 4 JUNIA-HEI (Hautes Études d’Ingénieur), F-59000 Lille, France; 5 Imagerie Multimodale Multiéchelle et Modélisation du Tissu Osseux et articulaire (I3MTO), Université d’Orléans, F-45067 Orléans, France; 6 Lille Catholic Hospital (GHICL), F-59160 Lomme, France; poupart.julien@ghicl.net (J.P.); chieux.vincent@ghicl.net (V.C.)

**Keywords:** multiple sclerosis, gel electrophoresis, isoelectric focusing, oligoclonal bands, cerebrospinal fluid, tears, immunoglobulin G, band straightening, image warping

## Abstract

The detection of immunoglobulin G (IgG) oligoclonal bands (OCB) in cerebrospinal fluid (CSF) by isoelectric focusing (IEF) is a valuable tool for the diagnosis of multiple sclerosis. Over the last decade, the results of our clinical research have suggested that tears are a non-invasive alternative to CSF. However, since tear samples have a lower IgG concentration than CSF, a sensitive OCB detection is therefore required. We are developing the first automatic tool for IEF analysis, with a view to speeding up the current visual inspection method, removing user variability, reducing misinterpretation, and facilitating OCB quantification and follow-up studies. The removal of band distortion is a key image enhancement step in increasing the reliability of automatic OCB detection. Here, we describe a novel, fully automatic band-straightening algorithm. The algorithm is based on a correlation directional warping function, estimated using an energy minimization procedure. The approach was optimized via an innovative coupling of a hierarchy of image resolutions to a hierarchy of transformation, in which band misalignment is corrected at successively finer scales. The algorithm’s performance was assessed in terms of the bands’ standard deviation before and after straightening, using a synthetic dataset and a set of 200 lanes of CSF, tear, serum and control samples on which experts had manually delineated the bands. The number of distorted bands was divided by almost 16 for the synthetic lanes and by 7 for the test dataset of real lanes. This method can be applied effectively to different sample types. It can realign minimal contrast bands and is robust for non-uniform deformations.

## 1. Introduction

### 1.1. Clinical Context

Multiple sclerosis (MS) is still an incurable disease and constitutes the main cause of non-traumatic disability in the young adult. Early diagnosis of MS enables the initiation of disease-modifying therapies that reduce the occurrence of relapses and slow disease progression. There is no specific diagnosis test for MS; a number of clinical, imaging, and laboratory criteria evidence the spread of demyelinating, inflammatory MS lesions in space and over time.

Analysis of cerebrospinal fluid (CSF) has been a valuable diagnostic tool for MS since Poser et al. published their criteria in 1983 [1]. Although an inflammatory CSF profile is not specific for MS, the intrathecal synthesis of immunoglobulin G (IgG) antibodies is strongly suggestive of MS once other causes of central nervous system inflammation have been ruled out.

The combination of isoelectric focusing (IEF) with IgG immunoblotting is the gold-standard quantitative assay for specific IgG oligoclonal bands (OCBs) in CSF and serum [2]. CSF proteins and serum proteins are focused on their isoelectric points through the joint action of a vertical pH gradient and a powerful electric field. Paired CSF and serum IEF profiles are examined to search for at least two thin, horizontal OCBs that are present in the CSF but not in the serum. The latest revision of the McDonald criteria emphasized the role of CSF-specific OCBs, which can replace the need to demonstrate a dissemination in time for patients with a first clinical event (“clinically isolated syndrome” (CIS)) [3].

Examining CSF for OCB detection requires a lumbar puncture—a relatively invasive and painful procedure. Previous research by our group demonstrated a good level of agreement between OCBs in CSF and those in non-invasively extracted tear samples and thus suggested that tears can be used as the first-line samples for OCB detection [4,5].

### 1.2. The Automatic Analysis of IEF Membranes

**Figure 1:**
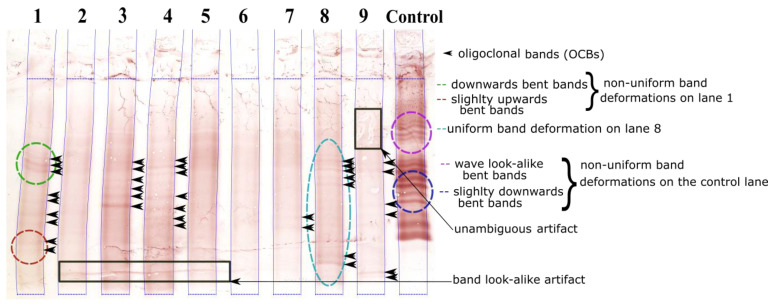
Example of a CSF IEF membrane with different types of band deformation: the left and right contours of each lane are delineated (blue lines) using our automatic lane segmentation method described in [11].

Figure 1 shows an example of an IEF gel membrane with CSF samples from nine patients. At present, each lane on an IEF membrane is inspected by eye for OCBs; hence, the results depend strongly on the quality of the membrane and are subject to inter-rater variability [6]. In fact, the IEF membranes often contain types of artifacts that can be mistaken for OCBs (Figure 1). Furthermore, samples with a low IgG concentration gives low-contrast profiles with faint bands that are hard to distinguish visually.

An automatic tool would (i) speed up IEF membrane analysis and OCB detection considerably, (ii) remove inter-user variability, (iii) enable the detection of bands obscured in the lane background, and (iv) facilitate the analysis of complex, noisy profiles.

To the best of our knowledge, there are no suitable tools for the automatic analysis of IgG IEF membranes. In fact, most of the available electrophoresis image analysis tools have been developed for DNA gels and are thus suited to the high contrast of DNA profiles and bands. Bajla et al. [7,8] described a full solution for IEF of erythropoietin (EPO). However, EPO bands on IEF gels are revealed by a chemiluminescence reaction, and the IEF membranes have high-contrast profiles and bands that are easily distinguished from the background. Hence, Bajla et al.’s software is not suitable for IgG IEF analysis.

We are currently developing the first automatic tool for OCB detection in CSF and tear samples [9,10,11]. Our objective is to provide an easy-to-use solution with a reasonable processing time, to ensure its adoption by hospital physicians.

### 1.3. Geometric Band Distortions in IEF Images

Small variations in experimental conditions during protein migration (an uneven heat distribution, variations in the electric field, etc.) often result in geometric distortion in IEF membranes. Firstly, the ideally vertical, straight lanes are deformed and have a vertical, “smile-like” shape (e.g., lane 1 in Figure 1). Secondly, the ideally horizontal straight bands are also deformed (e.g., the bands in lanes 1, 8 and control in Figure 1).

Correcting geometric distortion and hence restoring ideal IEF images are the first steps in the automatic analysis of IEF membranes. Our previous work addressed the automatic segmentation of distorted lanes [11]; in Figure 1, the blue lines correspond to the segmentation results for each lane. Here, we address the problem of distorted bands in IEF images.

**Figure 2:**
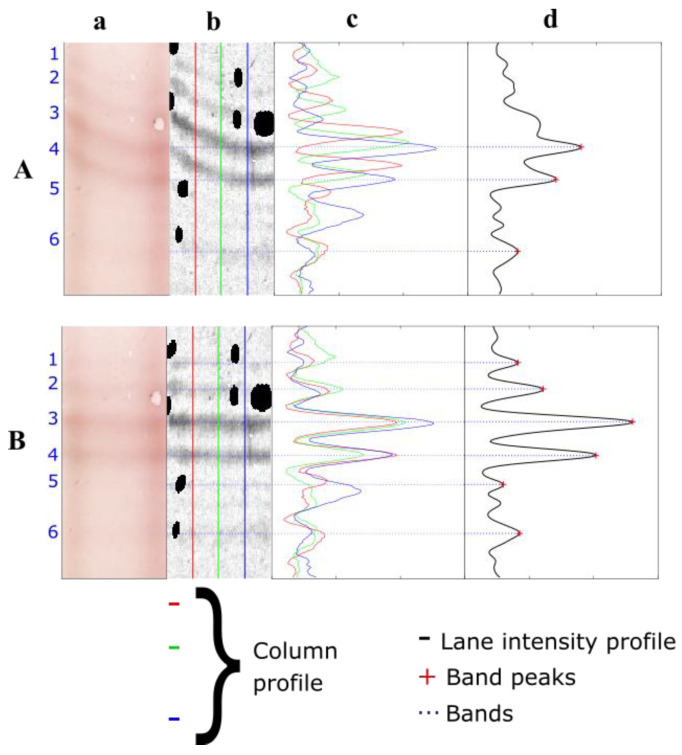
Illustration of the importance of band straightening for faint, non-horizontal bands in a CSF lane: (**Aa**): a lane with deformed bands; (**Ba**): the same lane with straight bands; (**Ab**,**Bb**): a background-subtracted grayscale image (contrast ×12, for clarity); (**Ac**,**Bc**): three column profiles with and without band deformations, band peaks and signal valleys are aligned in (**Bc**); (**Ad**,**Bd**): a 1D intensity profile. The band peak amplitudes in (**Bd**) are higher than those in (**Ad**). Non-horizontal, low-intensity bands 1, 2 and 5 are not detectable on (**Ad**) but are detectable on (**Bd**).

Band deformation might dramatically affect the performance of an OCB detection algorithm. Indeed, given that OCBs are narrow peaks above a defined threshold on the one-dimensional (1D) intensity profile of each lane (i.e., the average of all columns) [9], non-horizontal bands have broader peaks and lower amplitude (due to contrast spreading) (Figure 2). This occurs frequently for low-intensity profiles of tear samples and is problematic for low-contrast bands drowned in the background. Hence, band straightening is a crucial step in the recovery of undetected bands (Figure 2(Aa,Ad,Ba,Bd)).

We estimate that about 10% to 20% of IEF profiles have band deformations that reduce the reliability of OCB detection. Typical band deformation can be classified as uniform or non-uniform (see Figure 1). Uniform deformations feature the same shift in each given column. For non-uniform deformations, the shifts in each given column differ according to the height in the lane.

### 1.4. Related Research on Band Straightening

Our group developed an initial version of an OCB-straightening algorithm for IgG IEF images in 2016 [9]. It was based on finding the vertical displacement vector that maximized the correlation between image columns. However, since the same vertical shift is present in each given column, the algorithm could not remove band distortions in lanes with non-uniform band deformations. A similar approach has been described by Stolc and Bajla [8] for band straightening in EPO IEF images. Nonetheless, the search for the displacement vector was limited to relevant columns in the central region of the lane.

A realignment warping algorithm based on optimizing the correlation between corresponding segments in reference and target images has been used to correct for misalignment when registering and comparing chromatographic data [12,13]. Moreira et al. [14] used a similar algorithm to correct the geometric distortion of bands in thin layer chromatographic images. Their method is based on maximizing the correlation between each lane column and a reference column averaged from the central region of the image. Moreira et al. usefully address non-uniform deformation by applying a different, independent warping function to each group of bands identified as local maxima in the lane intensity profile. However, given that each region is treated independently, the resulting overall deformation lacks smoothness and continuity. Furthermore, the algorithm will fail if the central region (and then the reference column) is contaminated with artifacts.

Vauterin et al. [15] described a band realignment method for DNA gel electrophoresis images based on running a control sample between samples at regular intervals. The vertical shift needed to align each control sample band in order to determine the band positions of the reference sample. This method is impossible to test on our images because only one control sample is run on an IgG IEF membrane.

Several semi-automatic approaches for straightening deformed bands in DNA gel electrophoresis images have been described in the literature [16,17]. The main idea behind these approaches is the mapping of anchor lines or anchor bands drawn by the user to straight horizontal lines, followed by the linear interpolation of the intervening gel image region to correct the geometric distortion of the bands.

Here, we describe a novel, fully automatic, computationally efficient, reliable band-straightening algorithm that is suitable for all classes of band geometric distortion on IEF of CSF, serum, and tear samples. Our approach was implemented through optimized hierarchical warping and then tested on digitally simulated and real IEF lanes.

## 2. Materials and Methods

### 2.1. Description of the Datasets

#### 2.1.1. IEF Image Acquisition and Pre-Processing

IEF lanes of CSF, serum, and tear samples were acquired during the POLAR French national multicentre clinical trial. POLAR was conducted from 2012 to 2018 and was coordinated by our biologists from the biochemistry laboratory at Saint Philibert Hospital (Lille, France). The trial was designed to assess the diagnostic value of OCBs detected in IEF gels of tear samples from patients with a CIS. Tears, CSF and serum samples were collected for each patient to study the relationship between tears/CSF IEF. IEF and IgG immunoblotting were carried on a 10 cm × 8 cm gel membrane, using the Helena Biosciences IgG IEF kit intended for IgG OCB detection in CSF and serum, and using a SPIFE 2000 analyser (Helena Biosciences Europe, Gateshead, UK).

Dried membranes were then scanned at a resolution of 600 dpi (V750 PRO scanner, Epson, Levallois-Perret, France) and stored in a 24-bit jpeg format. Hence, the typical image size is 2380 × 1905 pixels. Typical IEF images contain up to nine vertical strips (also called lanes or profiles) corresponding to the same sample type (tears, serum or CSF but generally from different patients) and an additional control sample in the 10th (or right-hand-most) lane (Figure 1).

The RGB images were linearly transformed into grayscale images using the equation (0.16 × R + 0.52 × G + 0.32 × B) described in [9]. The scanned images were then cropped vertically to remove the blank sample-free margins (2.12 cm (505 pixels) at the top of the image and 0.42 cm (100 pixels) at the bottom). Each lanes’ left and right edges ($x_{right}\left(y\right)\;,x_{left}\left(y\right))$ were simultaneously delineated using our innovative automatic lane segmentation approach, which was developed for highly distorted and low-contrast lanes [11]. This approach is a new formulation of the classic parametric active contour problem, in which an open active contour is constrained to move from the top to the bottom of the image and the x-axis coordinate is expressed as a function of the y-axis coordinate (lane segmentations are shown in blue lines in Figure 1).

Each segmented lane was then limited to an automatically detected region of interest (ROI), corresponding to the IgG migration zone on the gel membrane (the horizontal blue lines at the top and at the bottom of each lane in Figure 1).

Next, each segmented lane was rectified to a perfectly vertical profile of width $w_{max}$, corresponding to the maximum width difference between the left and right edges: $w_{max}=\underset{y}{\max}\left(x_{right}\left(y\right)-x_{left}\left(y\right)\right)$. The lane was straightened by realigning a virtual line that joins the middle pixels between the lane edges for each row. Empty borders were considered as missing values. Unambiguous artifacts (i.e., not band look-alikes) were finally ruled out (by labelling them as missing values) using the algorithm described in [9], so that the band-straightening algorithm was not influenced by artifacts. The results of the last two pre-processing steps will be shown later in the manuscript (Section 2.3.1).

The geometric deformation affecting each pixel with coordinates (x,y) in the lane image is a combination of a vertical shift ΔY(x,y) (in pixels) and a horizontal shift ΔX(x,y). By the end of the pre-processing step, the horizontal deformation had been corrected and so the band-straightening algorithm was reduced to that of finding the vertical deformation ΔY(x,y).

#### 2.1.2. The Real IEF Dataset

A database of 150 lanes (50 each for CSF, tears, and serum) was distributed equally between training and test datasets. The lanes were randomly selected from the IEF database compiled during the POLAR multicentre clinical trial. IEF profiles that could not be analysed clinically (because of insufficient protein levels and thus insufficient contrast) were excluded from the database. Moreover, IEF lanes with strong artifacts that prevented visual analysis were also excluded.

Due to their right-most position in the gel membrane, control samples frequently showed strong geometric band deformations (Figure 1). Hence, 50 control lanes (25 in the test set and 25 in the training set) were added to the dataset.

The training set was used to optimize the values of the algorithm’s parameters, whereas the test set was used to assess the performance of the new algorithm.

#### 2.1.3. The Synthetic Dataset

One thousand digitally generated lanes were created to test our band-straightening algorithm’s ability to correct random artificial deformations.

The synthetic lane background was generated by randomly choosing one of five real negative grayscale profiles (i.e., profiles without OCBs) filtered beforehand with a two-dimensional (2D) Gaussian filter. Gaussian functions are commonly used in the literature to approximate band shapes [10,18] hence, the bands were created using a Gaussian density function. The expected value was the band position, and the standard deviation (SD) was proportional to the band width (band width ≈ 4 SD). The band position was a uniformly distributed random number in the lane height. The band’s full width at half maximum (FWHM) is set to 7 px+r, where r is a random variable with an exponential distribution and an expected value of 7 pixels (0.30 mm). The minimum FWHM (7 pixels (0.30 mm)) and the average FWHM (14 pixels (0.59 mm)) were therefore representative of real-world bands.

A random number of bands drawn from the discrete uniform distribution between 1 and 12 were placed at arbitrary positions on the lane height.

A random vertical shift was then applied to each pixel in the image. The overall deformation field was filtered with a large 2D Gaussian kernel to emulate smooth, non-uniform deformations on real IEF lanes. Black circles (i.e., missing values) with a random radius were distributed randomly all over the lane, in order to simulate unambiguous artifacts. Lastly, speckle noise was added to simulate the small, granular irregularities in a typical IEF lane background.

Examples of two digitally generated lanes and the digital addition of non-uniform band deformations are shown below—in Section 3.1.2. 

### 2.2. Background Removal

Since pixel intensity contains a mixture of background and foreground information, we chose to remove the background prior to band straightening. Moreover, background subtraction unveils faint bands in low-intensity profiles.

The rolling ball technique is a morphological approach to background removal and the correction of non-uniform brightness. It is often applied to gel electrophoresis images [15,19] and light microscopy images [20]. A sphere with a chosen radius is rolled through the 3D landscape representation of the image. A background-subtracted image is obtained by removing the structures the sphere rolls into and keeping the remaining structures.

**Figure 3:**
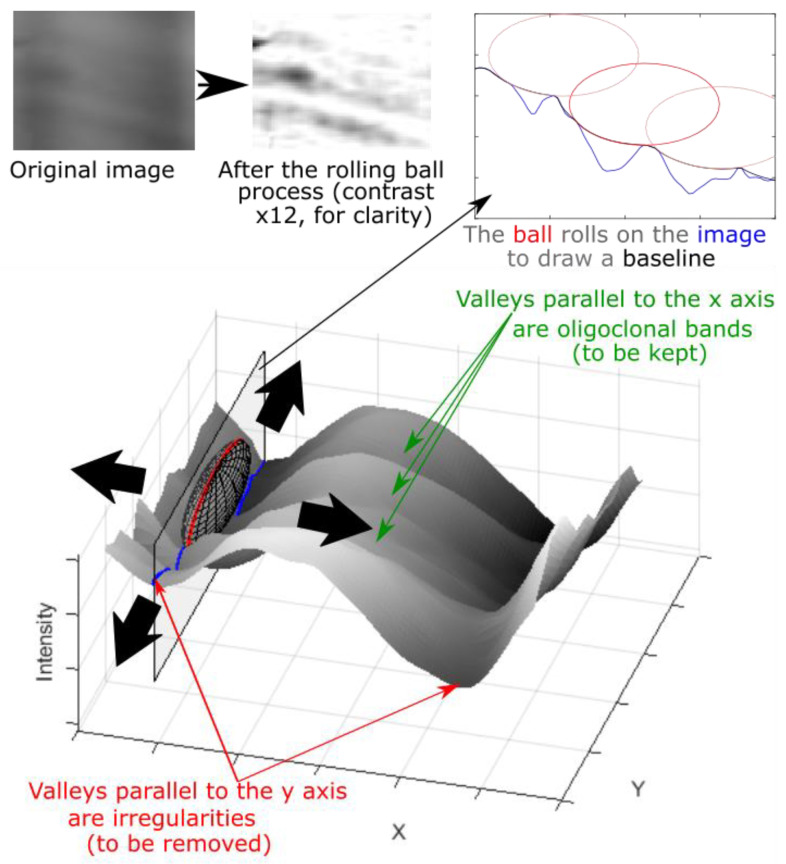
Illustration of the rolling ball approach for background and vertical irregularities subtraction: the ellipsoid falls into the valleys to be removed and rolls over the valleys to be preserved.

In our approach (Figure 3), an ellipsoid with radii $\left(r_{x}\;,r_{y}\;,r_{z}\right)$ was rolled over the 3D surface of the lane image. $r_{z}$ is the radius on the image’s z intensity axis (Figure 3). $r_{y}$ is chosen to be large enough to prevent the ellipsoid rolling into the band valleys parallel to the x-axis, which thus avoids band peak removal or attenuation. $r_{x}$ is determined so that the ellipsoid falls deep into valleys parallel to the y axis (corresponding to background variation). As the ellipsoid rolls over the lane surface and touches the background pixels, the background-free image is created by subtracting the obtained background image. Figure 3 schematizes the above process.

### 2.3. Correlation-Based Image Warping

Image warping is a transformation function f that maps the pixels with coordinates (x,y) from an original image $I_{0}$ to their corresponding coordinates $\left(x^{\prime },y^{\prime }\right)$ in a target image $I^{\prime }$ $\left(I^{\prime }\left(x^{\prime },y^{\prime }\right)=I_{0}\left(x,y\right)\right)$ [21]. The type of transformation (linear, affine, or elastic) is chosen a priori.

Image warping is often used to register multimodal medical images, to correct the optical distortion introduced by camera lenses, and to morph images [21]. In 2D gel electrophoresis image analyses, image warping is used to align several profiles for comparison or align a profile with a reference gel for band matching. It is also used to correct for geometric distortion in gels [14,16,17].

Semi-automatic warping approaches are guided by a set of matching landmarks placed by the user on the original and target images. The automatic approaches find the suitable transformation functions by making use of similarity measures of the original and target images. Similarity measurements can be based on pixel intensity, the correlation between corresponding parts in the original and target images, or similar features detected automatically in the original and target images.

Nielsen et al. developed a correlation-based image warping algorithm [12] to align 1D chromatographic traces. A modified version of this method has been used to straighten bands on thin layer chromatography images [14].

However, and as mentioned in Section 1, these correlation-based image warping approaches lack the smoothness and the continuity needed to address the band-straightening problem in IEF images. In image warping problems, the deformation elastic potential is often chosen as the non-smoothness penalty term [21]. Hence, we decided to build an improved correlation-based image warping algorithm that maximized the lane column correlations while penalizing strong or non-smooth deformations. The image warping problem for band straightening can therefore be described as a search for the optimal deformation that minimizes a cost function by finding a compromise between (i) a good match between the columns in the lane image and (ii) a limited, smoothed transformation.

#### 2.3.1. The Energy Minimization Approach for Image Warping

Energy minimization approaches have been used [22,23] to derive image warping functions: a set of allowed image deformations were evaluated and then kept or dismissed, depending on their contribution to minimization of the energy function.

As the horizontal geometric distortion had already been corrected (Section 2.1.1), the image warping problem for band straightening is restricted to deformations in the y axis. This implies that the pixel with coordinates (x,y) in the original lane image $I_{0}$ is transformed to $\left(x,y^{\prime }\right)$ in the warped lane image $I^{\prime }$, where $y^{\prime }=y+\Delta Y\left(x,y\right)$. The relationship between the original lane image and the warped one is therefore as follows:

(1) $$I^{\prime }\left(x,y+\Delta Y\left(x,y\right)\right)=I\left(x,y\right)$$

Our band-straightening problem was formulated as the search for image deformations ∆*Y* that minimized a linear combination of two energy terms defined on the basis of a priori knowledge: an image-related external energy term $E_{ext}\left(\Delta Y;\;I\right)$ and a deformation-penalizing internal energy term $E_{int}\left(\Delta Y\right)$.

- External energy

For a given lane with perfectly straight horizontal bands, the hills and valleys of individual column profiles had coincident positions (Figure 2(Bc)). Band peaks were thus well preserved, and their amplitude was maximized in the lane’s average 1D pixel intensity profile (Figure 2(Bd)). However, for a lane with geometric distortions of bands, the hills and valleys of column profiles were shifted (Figure 2(Ac)); this has the unfortunate effect of producing lower band peak amplitudes and, thus, OCB detection errors (Figure 2(Ad)). Hence, restoring each lane column’s correlation with the other columns would conserve band peaks and reveal faint non-horizontal bands for more reliable automatic detection of OCBs. The external energy term is then defined as:

(2) $$E_{ext}\left(\Delta Y;I\right)=f\left(\sum_{i,j\;\left(i\neq j\right)}R_{i,j}\left(\Delta Y;I\right)\right)$$

where i, j are lane columns indices ranging from 1 to the maximum lane width $w_{max}$, and $R_{i,j}$ is the (i,j) element in the correlation matrix R of the columns in the warped image $I^{\prime }$. f is a decreasing function with f(0)=1 and f(1)=0. f is chosen to give greater weight to the external energy term (relative to the internal energy weight), depending on the average column correlation. Hence, stronger deformations were allowed for profiles with multiple bands (and thus greater correlations).

Since the band-straightening method was applied to filtered lane images in which unambiguous detected artifacts are coded as missing values (Section 2.1.1), $\;R_{i,j}$ was computed only for lane rows where both columns i and j were not equal to missing values. This prevented artifacts from biasing the correlation maximization process.

b. Internal energy

**Figure 4:**
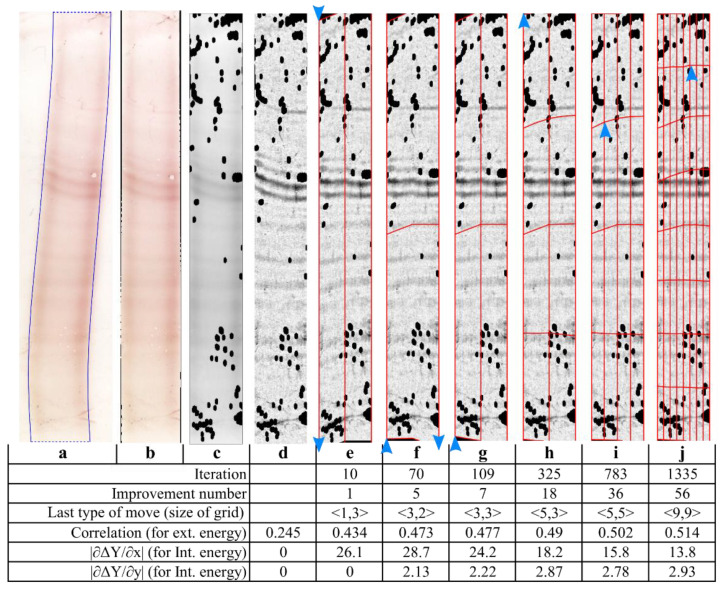
Illustration of the band-straightening method steps with a CSF lane: (**a**) lane segmentation, (**b**) lane straightening, (**c**) grayscale conversion and removal of unambiguous artifacts (the black zones on the lane), (**d**) Background removal (contrast ×12 for clarity), (**e**–**i**) are examples of intermediate steps chosen to illustrate the iterative process of band straightening with progressively finer moves (the deformed grid is superimposed on the image in red). Each blue arrow indicates the direction of the shift applied to the chosen grid point during the last iteration. (**j**) The final result.

The internal energy was based on a priori knowledge about the deformation type in a lane image. $E_{int}$ is a combination of two terms. The first term penalizes shear strain deformations. These deformations can be measured as the derivative of the vertical shift ΔY(x,y) divided by the x axis direction: $\frac{\partial \left(\Delta Y\left(x,y\right)\right)}{\partial x}$ (e.g., the left part of Figure 4e).

The second term penalizes dilation and compression deformations (i.e., normal strain deformations). Since our bands were deformed on the y axis direction only, we then evaluated normal strain deformations by measuring the derivative $\frac{\partial \left(\Delta Y\left(x,y\right)\right)}{\partial y}$ (e.g., the bottom left part of Figure 4g is compressed).

We then defined $E_{int}$ as:

(3) $$E_{int}\left(\Delta Y\right)=w_{x}\left|\frac{\partial \left(\Delta Y\right)}{\partial x}\right|+w_{y}\left|\frac{\partial \left(\Delta Y\right)}{\partial y}\right|\;$$

where $w_{x}$, $w_{y}$ are the weighting parameters and |.| corresponds to the sum of absolute values ($\left|g\right|=\sum_{x,y}\left|g\left(x,y\right)\right|$).

$E_{int}$ acts as a regularizer for the band-straightening problem because it tends to prevent strong, irregular deformations.

c. The deformation constraint

To avoid the compression of image regions with no bands and a low column correlation (e.g., the extreme upper and lower parts of the lanes in Figure 4) and the expansion of regions with a high column correlation (the middle part of the lanes in Figure 4), a deformation constraint ∫ΔY(x,y)dx=0  was added to the energy minimization problem. The constraint ensured that the average vertical shift for each image row is null.

The final energy minimization problem was as follows:

(4) $$\Delta Y^{*}=\underset{\Delta Y}{\mathrm{argmin}\;}E\left(\Delta Y;\;I\right)=\underset{\Delta Y}{\mathrm{argmin}}f\left(\underset{i,j\;\left(i\neq j\right)}{\sum^{\;}\;}R_{i,j}\left(\Delta Y;I\right)\right)+w_{x}\left|\frac{\partial \left(\Delta Y\right)}{\partial x}\right|+w_{y}\left|\frac{\partial \left(\Delta Y\right)}{\partial y}\right|,\;\mathrm{subject}\;\mathrm{to}\;\int \Delta Y\left(x,y\right)dx=0$$

#### 2.3.2. The Transformation Hierarchy

Searching for the optimal deformation field for every pixel that minimizes the overall energy is a high-dimensional minimization problem.

To increase the computational efficiency, gel image warping techniques rely frequently on a hierarchical search for transformations [23,24,25]. Hence, a gel image can be first partitioned into a set of large segments (triangles, rectangles, etc.) and an overall, coarse transformation is then rapidly built from piecewise local transformations [26]. Segments are then subdivided into smaller partitions, and the image warping transformations are refined successively.

Hence, we decided to build the overall lane warping function from successive finer local transformations, based on a hierarchical rectangular deformation grid ($r_{g}$ rows × $c_{g}$ columns). We defined a type of move $M_{g}$, corresponding to each deformation grid $\langle r_{g},c_{g}\rangle$. A move of type $M_{g}$ consists of choosing a grid point k with coordinates $\left(x_{k},\;y_{k}\right)$ belonging to the grid $\langle r_{g},c_{g}\rangle$ and in moving it up or down with a vertical shift $\delta^{k}$. The vertical shift ΔY(x,y) for each other pixel (x,y) was built by linear interpolation between grid points. Prior to the interpolation, the grid points in the same row as k were slightly moved in the opposite direction to ensure that ∀y, ∫ΔY(x,y)dx=0. The warped image $I^{\prime }$ was built next by applying the deformation ΔY (Equation (1)). If ΔY had successfully reduced the overall energy function, it was kept; if not, it was discarded.

First, a coarse deformation grid was superimposed on the lane image (Figure 4e). The grid was consecutively refined by adding rows or columns, which therefore allowed more local deformations (Figure 4i,j). Moreover, the vertical shifts of grid points were progressively reduced to reach a fine-scale solution. The warping process ended when the energy was no longer minimized.

#### 2.3.3. Coupling of a Hierarchy of Image Resolutions to a Hierarchy of Transformations

Multiresolution image strategies reduce the computation time of image-warping algorithms without decreasing the robustness of the resulting transformation. Coupling a hierarchy of images at different resolutions to a hierarchy of transformations has been applied previously to 2D gel image registration [23].

In our band-straightening approach, a coarser grid level was combined with lower resolution images and vice versa. A rough geometric image warping function was first obtained at coupled coarse grid and image resolutions. Then, as the iterations increased, the resolution incremented in the image and in the deformation spaces, until the desired finest solution was reached.

Since the image column correlation is the most time-consuming step and its complexity is O($c^{\alpha }\;r$) (where c is the number of columns, r is the number of rows, and 1<α<2 is a constant dependent on the matrix multiplication algorithm used [27]), it is judicious to choose a different multiresolution factor for lane columns $f_{c}$ and lane rows $f_{r}$ with $f_{c}>f_{r}$, in order to make the correlation computation more efficient.

Our multiresolution approach was therefore divided into four hierarchical steps j=1… 4, with their respective pairs of row and column scale factors ($f_{r_{j}},f_{c_{j}})=\left(\left(8,8\right),\;\left(4,8\right),\;\left(2,8\right),\;\left(1,4\right)\right)$. Further refinement of the image resolution did not improve the band realignment. Moreover, we linked each hierarchical step j to a vertical shift value $\pm \;\delta_{j}$ starting at $\delta_{1}$ and decreasing for every j to reach $\delta_{4}$ at the finest image resolution.

#### 2.3.4. The Band-Straightening Algorithm

Below, we describe the entire algorithm:

Begin with ΔY(x,y)=0

For a hierarchical step j=1…4 linked to a vertical shift $\delta_{j}$:

- Downsample the original lane image $I_{0}$ with the corresponding scale factors ($f_{r_{j}},f_{c_{j}})$ (Section 2.3.3)
- Apply the deformation ΔY to obtain the current image: $I\left(x,y+\Delta Y\left(x,y\right)\right)=I_{0}\left(x,y\right)$
- For each type of move $M_{g}\;\in \;{ℳ}_{j}$ (${ℳ}_{j}$ being a list of types of move specific for each hierarchical step j) corresponding to a grid size $\langle r_{g},c_{g}\rangle$:
  - Choose a random permutation $L_{g}$ of the set of the $r_{g}\times c_{g}\;$ grid points
  - For each grid point k in $L_{g}$ with coordinates $\left(x_{k},y_{k}\right)$ and for each possible sign s ∈ {−1,1} of the shift (−1 being down, and 1 being up):
    1. Move vertically k with $\delta^{k}=s\times \delta_{j}$
      $$\Delta Y^{\prime }\left(x_{k},y_{k}\right)=\Delta Y\left(x_{k},y_{k}\right)+s\times \delta_{j}$$
    2. Adjust the position of grid points on the same row as k, to comply with the band-straightening algorithm’s constraint $\int \Delta Y^{\prime }\left(x,y\right)dx=0$ (Section 2.3.2)
    3. Linearly interpolate between grid points to obtain the warped lane image $I^{\prime }$
    4. If $E\left(\Delta Y^{\prime };I^{\prime }\right)<E(\Delta Y;I$):
      (a) Put the 4-neighbor grid points of k at the end of $L_{g}$ (except when k is located at border)
      (b) Move k  with a smaller shift (equal to $0.5\times s\times \delta_{j}^{y}$) and a larger shift (equal to $1.5\times s\times \delta_{j}^{y}$) and repeat steps 2 and 3 to obtain $\left(I_{0.5}^{\prime },\Delta Y_{0.5}^{\prime }\right)$ and $\left(I_{1.5}^{\prime },\Delta Y_{1.5}^{\prime }\right)$
      (c) Set $\left(I,\Delta Y\right)=\underset{\left(I,\Delta Y\right)\in \left\{\begin{array}{c} \left(I_{0.5}^{\prime },\Delta Y_{0.5}^{\prime }\right)\\ \left(I^{\prime },\Delta Y^{\prime }\right)\\ \left(I_{1.5}^{\prime },\Delta Y_{1.5}^{\prime }\right) \end{array}\right\}}{\mathrm{argmin}}E\left(\Delta Y;\;I\right)$

Figure 4 shows the change in energies during the chosen illustrative intermediate steps. At the beginning (Figure 4d), the external energy is high because the columns are weakly correlated, and the internal energy (represented by the two partial derivative terms) is null, since no geometric corrections have yet been performed. After a few iterations (Figure 4e,f), the external energy decreased dramatically (since the column correlation increased) but the internal energy increased (since the lane was being deformed). A rough solution was then achieved. Further iterations led the external energy to fall slightly (as the column correlation increased) but reduced the internal energy (mostly by smoothing the lanes’ overall deformation).

#### 2.3.5. The Algorithm’s Settings

Algorithm parameters listed herein (the rolling ellipsoid radii $\left(r_{x}\;,r_{y}\;,r_{z}\right)$, the decreasing function  f in $E_{ext}\left(\Delta Y;I\right)$, the $E_{int}\left(\Delta Y\right)$ weighting parameters $w_{x}$, $w_{y}$, the shift $\delta_{j}$ associated with each hierarchical step j, the list of types of move ${ℳ}_{j}$ specific for each hierarchical step j) are not disclosed here due to a technology transfer being negotiated. All the latter parameters were adjusted and set empirically through trial-and-error experiments on the training dataset of real and synthetic lanes.

#### 2.3.6. Optimization of the Band-Straightening Algorithm

Our approach uses powerful optimization techniques to produce a low-cost, reliable band-straightening method. The coupled hierarchy of image resolutions and hierarchy of transformations gave much the same results as the hierarchy of transformations alone (in the absence of a multiresolution framework), but led to a decrease in the computational cost by a factor of ~3.

Most of our methods for the automatic analysis of IEF images and the detection of OCBs were developed in MATLAB. However, the time-consuming parts (image warping, correlation calculation) were implemented in C/C++, using the MKL routines (version 2020.1). This latest time optimization method has reduced the computation time by 20%, relative to the MATLAB version.

Hence, the computation time for a full, 10-lane membrane is approximately 10 s when each lane is processed on a different thread (Intel I7-7700HQ laptop computer, MATLAB R2020b). This computation time is appropriate for a fluent user experience.

### 2.4. Evaluation Methods

#### 2.4.1. Real IEF Dataset

We used a rigorous method to evaluate our band-straightening algorithm. Firstly, we developed an interactive graphical user interface for expert manual band annotation. One expert analysed the training and test datasets, and a second expert reviewed the analysis. Bands were manually traced on the original lane images, i.e., without automatic band straightening. The experts placed points on the band’s midline, which were then linearly interpolated. To guide the manual annotation process, the tracing assistant tool generates instant feedback on the successive points placed by the expert. This feedback consists of showing a warped lane image by mapping the lines traced by the expert to horizontal lines. Thus, the expert can check if he/she has correctly annotated the bands by checking whether they are straight on the warped image. This tool is particularly important for large bands with spread contrast and for bands with faint borders, on which the band’s midline is ambiguous.

We used different criteria to assess the performance of our band-straightening approach on profiles with or without bands. For profiles without bands, we measured the amount of unnecessary deformation introduced during the band-straightening process. For profiles with at least one band, we measured the degree of deformation of the bands delineated by the expert before and after automatic band-straightening.

The deviation from a horizontal straight band was measured as the SD: a horizontal band will have an SD of zero. Thus, if the band drawn by the expert is expressed as y(x), we measured the SD before straightening ($SD_{x}\left(y\left(x\right)\right)$ and after straightening $\left(SD_{x}(y\left(x\right)+\Delta Y\left(x,y\left(x\right)\right)\right)$.

Comparing the degree of deformation of bands with an SD below 1 pixel before and after band straightening was irrelevant, since (i) the intra-user variability of user placed bands was estimated to be more than 1 pixel and (ii) a 1-pixel deformation is negligible and would not affect the band peak detection (the OCB width (FWHM) ranges from about 8 pixels (0.34 mm) to 30 pixels (1.27 mm)).

Hence, the effectiveness of band straightening was evaluated quantitatively by comparing the number of bands before band straightening ($num_{before}$) and after band straightening ($num_{after}$) with a degree of deformation greater than the four chosen thresholds (t=2,3,4 or 5 pixels):

(i) SD > 2 pixels correspond to a negligibly to-strongly deformed band.
(ii) SD > 3 pixels correspond to a weakly to-strongly deformed band.
(iii) SD >4 pixels corresponds to a moderately-to-strongly deformed band.
(iv) SD > 5 pixels correspond to a strongly deformed band.

Strongly deformed bands included in (iv) will also be included in (i), (ii), and (iii).

The algorithm’s overall performance for band profiles was quantified by measuring the ratio $\rho_{t}=\frac{num_{before_{t}}}{num_{after}{}_{t}}$ with the different deformation thresholds t.

For profiles without bands, the degree of deformation introduced was evaluated by calculating the average SD of all image rows after application of the band-straightening algorithm. Next, the individual deformations were averaged for all the profiles without bands in the training and test datasets separately.

We compared the performance of the latest OCB-straightening algorithm (OCBSA-2021) with that of a correlation-based band straightening algorithm previously developed by our group (referred to here as OCBSA-2016) [9]. The results were specified for each type of sample (CSF, serum, tears, or control).

#### 2.4.2. Synthetic Dataset

As the ground truth is already known for synthetic profiles, the band-straightening algorithm can be judged objectively. The same SD-based evaluation method was used to evaluate the performance of OCBSA-2016 and OCBSA-2021 to correct digitally generated band deformations. 

## 3. Results and Discussion

### 3.1. Illustrative Results

#### 3.1.1. Results with the Real IEF Dataset

This section presents illustrative results on lanes selected for their difficulties of straightening.

**Figure 5:**
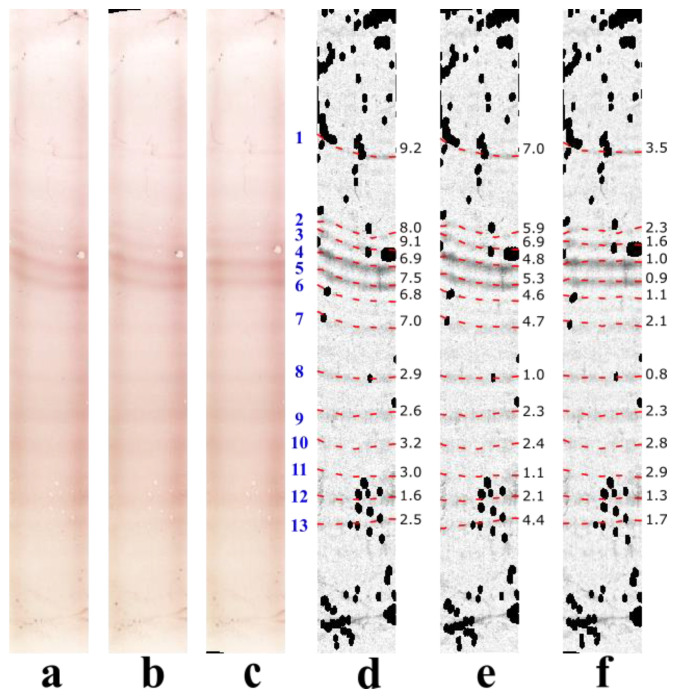
Results of band straightening on a CSF lane: (**a**–**c**): original colors; (**d**–**f**): grayscale converted, background subtracted, non-ambiguous artifact in black and contrast ×12 for clarity; (**a**,**d**): original deformation; (**b**,**e**): the output of OCBSA-2016; (**c**,**f**): the output of OCBSA-2021. Bands are shown as red dashed lines, and the corresponding SD are displayed on their right.

Figure 5 shows the results of band straightening on a CSF lane with non-uniform band deformations: the upper seven bands are severely bent downwards (average SD: 7.9 pixels; Figure 5a,d) and the remaining six bands are slightly deformed (average SD: 2.6 pixels) (Figure 5a,d). This example demonstrated the robustness of OCBSA-2021 with regard to non-uniform band deformations: all the deformed bands were straightened to a satisfactory extent (Figure 5c,f). In contrast, OCBSA-2016 clearly failed to straighten the 7 upper bands (Figure 5b,e). Moreover, bands 1, 2, 3, and 6 illustrated OCBSA-2021′s ability to straighten faint bands with large angle deformations and thus confirmed its value as a technique for enhancing IEF gel images prior to OCB detection. A visual examination of the band straightening results (bands 1, 2, 10 and 11) (Figure 5c,f) illustrated the imprecision of the expert’s band delineation, which resulted in the underestimation of visually satisfactory straightening by OCBSA-2021.

**Figure 6:**
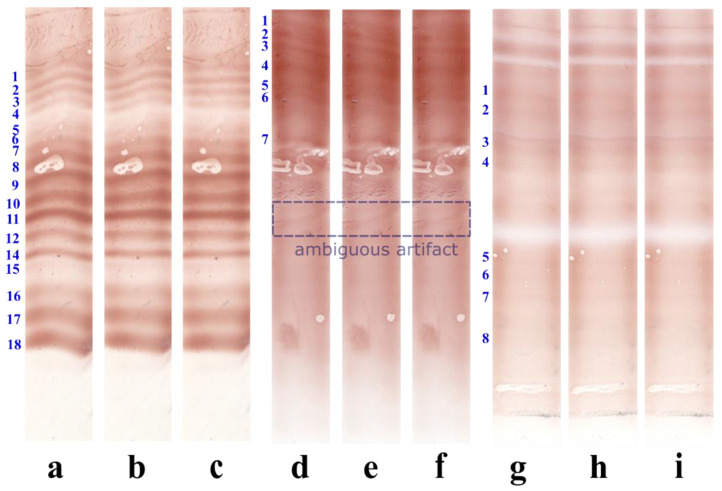
A comparison of band straightening with OCBSA-2016 (**b**,**e**,**h**) vs. OCBSA-2021 (**c**,**f**,**i**) on a control lane (**a**–**c**), a serum lane (**d**–**f**), and a tear lane (**g**–**i**).

Figure 6a,d show examples of control and serum lanes, respectively, with bands deformed in a non-uniform way. Figure 6b,e show examples of OCBSA-2016′s failure to straighten all the bands with non-uniform band deformations. In contrast, OCBSA-2021 gave excellent band-straightening results (Figure 6c,f). Figure 6i shows that OCBSA-2021 is also suitable for uniform band deformation distributions, as it successfully straightened the bands in the tear profile (Figure 6g). OCBSA-2021 and OCBSA-2016 gave similar results for this distribution of band deformations (Figure 6h,i).

Figure 6g is an example of a tear profile in which bands 5, 6, 7, and 8 are obscured by the lane background. Figure 6i demonstrates OCBSA-2021 ability to straighten bands with the low intensity levels frequently observed in tear profiles.

Figure 6a (band 18) and Figure 6d (the ambiguous, band look-alike artifact) contain deformations not straightened by OCBSA-2021 since the type of deformation was incompatible with the smoothness and the continuity characteristics of the local band deformations that can be observed in IEF lanes. The ambiguous artifact in Figure 6d was correctly ignored by OCBSA-2021 and was left in its deformed state (Figure 6f).

The rightmost border deformation of band 18 in Figure 6a,c reveals a minor limitation of OCBSA-2021. This abrupt, major deformation affected the band border but did not extend to the upper band and thus this part of the band was not straightened (Figure 6c).

**Figure 7:**
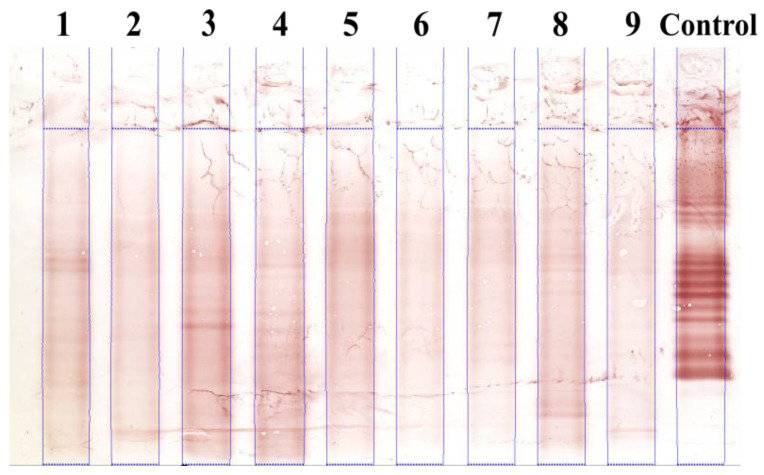
Illustration of a band-straightening result for the CSF membrane in Figure 1.

OCBSA-2021′s output for the entire CSF membrane in Figure 1 is displayed in Figure 7. Before processing, the profiles were mapped to ideal-looking, perfectly vertical lanes (Section 2.1.1). OCBSA-2021 successfully transformed the deformed bands in lanes 1, 8 and control (Figure 1) to ideal-looking horizontal bands. The ambiguous (band look-alike) artifact in Figure 1 was correctly ignored by the straightening algorithm in Figure 7.

#### 3.1.2. Results with the Synthetic Dataset

**Figure 8:**
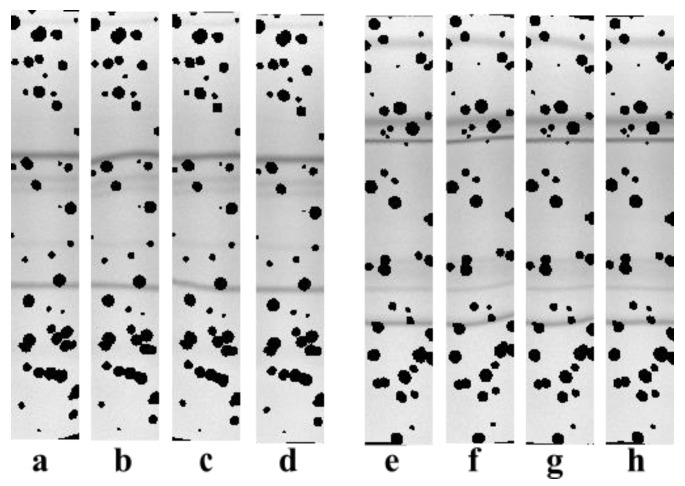
Illustration of band straightening on two synthetic profiles: (**a**,**e**): non-deformed synthetic lanes; (**b**,**f**): digitally added non-uniform band deformations; (**c**,**g**): results of band straightening with OCBSA-2016; (**d**,**h**): results of band straightening with OCBSA-2021.

Testing OCBSA-2021 on synthetic lanes exactly estimated the algorithm’s effectiveness, since the results obtained (Figure 8d,h) can be directly compared with the original, synthetic lanes with non-deformed bands (Figure 8a,e). Figure 8d,h illustrate OCBSA-2021′s ability (in contrast to OCBSA-2016) to straighten bands with a non-uniform deformation distribution (Figure 5c,g).

### 3.2. Statistical Evaluation of the Algorithm’s Performance with Real and Synthetic Data

**Figure 9:**
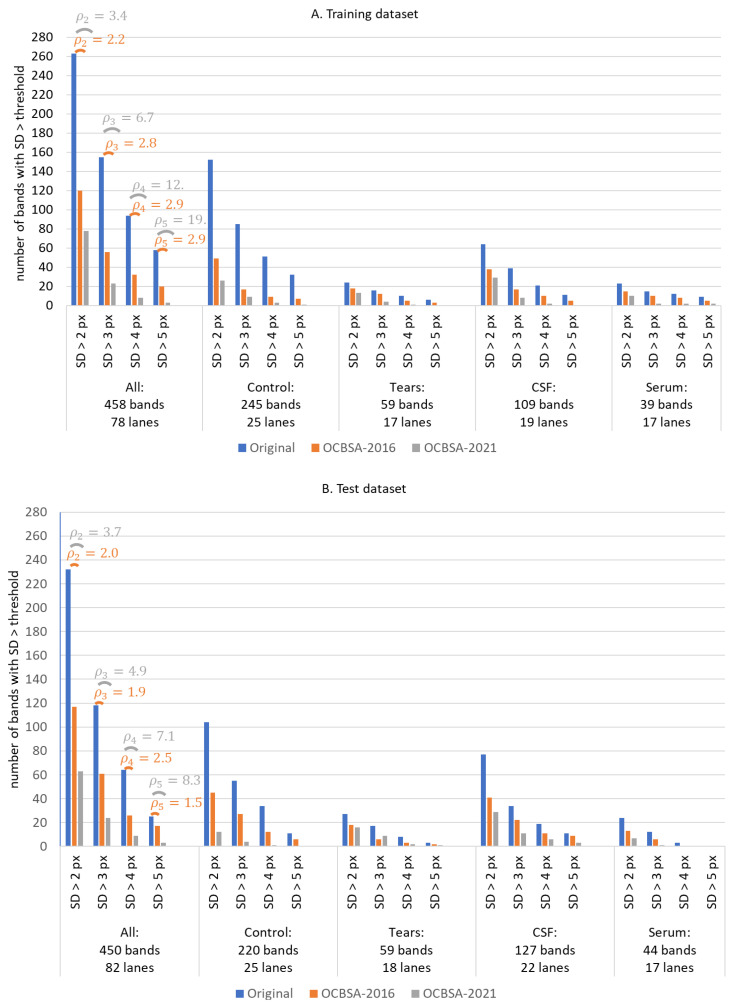
Band-straightening results for the real IEF training (**A**) and test (**B**) datasets: the number of bands with different deformation thresholds (SD > 2 pixels, 3 pixels, 4 pixels, 5 pixels) before straightening, after OCBSA-2016 and after OCBSA-2021 are displayed for the whole dataset and for each sample type (control, tears, CSF, and serum). The $\rho_{t}=\frac{num_{before_{t}}}{num_{after_{t}}}$ ratios with the different deformation thresholds are shown for the whole dataset (orange for OCBSA-2016, grey for OCBSA-2021).

The bar charts in Figure 9A,B summarize the quantitative results for OCBSA-2021 and OCBSA-2016 for profiles with a least one band from the training and test datasets. The detailed results for each sample type (CSF, serum, tears and control) are also illustrated. The number of bands with a deformation degree greater than t= 2, 3, 4 or 5 pixels is shown before band straightening (blue bars), after processing by OCBSA-2016 (orange bars) and after processing by OCBSA-2021 (grey bars). The ratio $\rho_{t}$ for the change in band number after band distortion correction is displayed for each threshold t and for the training and test datasets (orange: OCBSA-2016, grey: OCBSA-2021).

One can note the difference in the number of bands with SD (ΔY)>5 pixels between the training dataset (58 bands) and the test dataset (25 bands). This difference is mainly due to two control lanes with, respectively, 11 and 7 highly distorted bands randomly assigned to the training database during database decomposition. 

Serum lanes contain rarely bands; however, in order to study band straightening on this sample type, the experts chose to also annotate several faint bands with no real clinical value. Tear lanes have less bands than CSF lanes, and so there were twice as many CSF bands (training: 109, test: 127) than tear bands (training: 59, test: 59).

OCBSA-2021’s ability to decrease the number of deformed bands is obvious for large-angle, bent bands: in the training database, the number of strongly deformed band (SD > 5 pixels) was divided by $\rho_{5}$ = 19, and the number of moderately-to-strongly deformed band (SD > 4 pixels) was divided by $\rho_{4}$ = 12. In the test dataset, those factors for OCBSA-2021 are $\rho_{5}=$ 8.3 and $\rho_{4}=$7.1, respectively. These values were significantly better than those of OCBSA-2016: $\rho_{5}=\rho_{4}$ = 2.9 in the training database and $\rho_{5}=$ 1.5 and $\rho_{4}=$ 2.5 in the test database.

The difference in performance between training and test dataset is relatively small, which indicates the absence of overfitting with the chosen settings.

OCBSA-2021′s superiority over OCBSA-2016 comes from the fact that the latter was developed for small ROIs selected manually by experts inside each lane, and so the bands were unlikely to have a non-uniform deformation distribution. In our automatic approach, the analysed region is no longer a small ROI; the need for a non-uniform band deformation correction therefore arises, and OCBSA-2016 gives relatively poor results. 

OCBSA-2021 is operational for all profiles (serum, tears, CSF, and control) but is more effective on control and CSF lanes. This is due to OCBSA-2021′s relatively high sensitivity to band contrast: a low-contrast band is less likely to be perfectly straightened than a high-contrast band.

**Figure 10:**
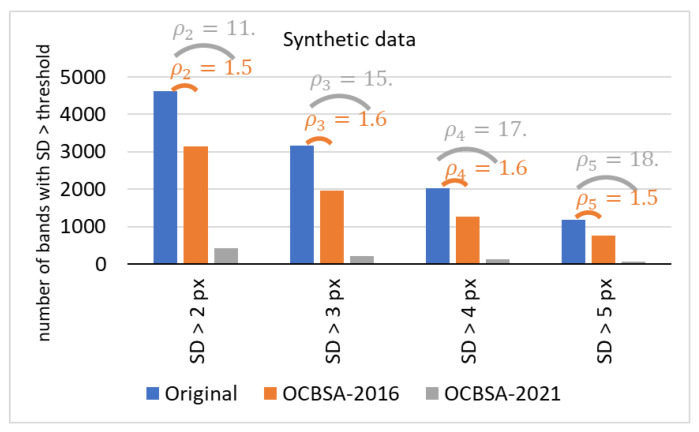
The band-straightening results for the synthetic dataset.

Figure 10 shows the results obtained for the synthetic lane database. Since the deformations created were non-uniformly distributed, the striking performance difference between OCBSA-2016 and OCBSA-2021 was expected. Relatively similar $\rho_{t}$ were obtained using OCBSA-2021 on the real and the digitally created lanes—especially for bands with SD > 4 pixels and bands with SD > 5 pixels. However, higher $\rho_{t}$ are observed for bands with SD > 3 pixels and bands with SD > 2 pixels—probably due to the perfect ground truth for this dataset.

Table 1 summarizes the results for profiles with no bands in the training and test datasets. A general decrease in the average introduced deformation SD for OCBSA-2021 vs. OCBSA-2016 was observed for the whole dataset. Both algorithms avoided creating unnecessarily strong deformations.

We demonstrated that our new band-straightening method OCBSA-2021 (i) is effective and reliable for correcting strongly deformed bands, (ii) surpasses OCBSA-2016 by decreasing the deformation by a factor $\rho_{t}$ ranging from 3.7 to 8.3 on the test dataset (depending on the considered degree of deformation), and (iii) successfully avoided unnecessary deformations on profiles with no bands.

A post hoc examination of the remaining deformed bands showed that most were due to imprecisions during expert band annotation or to broad bands that made it hard for experts to accurately locate the band’s midline. In this latter case, expert annotation-based straightening and the OCBSA-2021 solution both appeared to be acceptable, despite being significantly different. An improvement in column correlation was observed for all the profiles with at least one band from both the training and test datasets, relative to expert-annotation-based straightening. This finding implies that OCBSA-2021 always achieved a satisfactory solution and was never trapped in local minima.

### 3.3. OCBSA-2021’s Contribution to OCB Detection

An initial comparison of OCBSA-2021′s contribution to band detection vs. that of OCBSA-2016 was carried on 165 CSF lanes from the POLAR database, using an enhanced version of our previously published automatic OCB detection method [9]. The visual on-membrane expert consensus analysis was considered to be the ground truth. A profile with at least three OCBs was required to designate the profile as oligoclonal. The diagnosis was more precise with OCBSA-2021 (sensitivity: 0.88; specificity: 0.88) than with OCBSA-2016 (sensitivity: 0.86; specificity: 0.86). These improvements were probably due to the successful recovery of low-intensity bands with non-uniform deformation distributions. We intend to enhance our OCB detection method further.

## 4. Conclusions

Here, we described a novel band-straightening algorithm for the correction of geometric band distortion in IgG IEF images. The method has been tested on real IEF lanes and digitally created lanes. The results of our evaluation demonstrate that our approach is (i) effective for both uniform and non-uniform band deformation distributions (in contrary to our previous band-straightening algorithm [9]), (ii) functional for all tested sample types (serum, tears, CSF, or control), (iii) robust for low-intensity, background-obscured bands, and (iv) able to realign bands with different degrees of deformation.

The (y-axis) directional correlation-based image warping problem is formulated as the search for the overall deformation that minimizes a total energy equation. The external energy term is a function of the image’s column correlation, and the internal energy term is a regularizer that penalizes dilation/compression and shear deformations. The problem is optimized by coupling a hierarchy of image resolutions to a hierarchy of transformations; thus, optimal band straightening can be performed efficiently and at a low computation cost by using successively finer image and transformation scales.

The time-consuming parts of the algorithm have been optimized without sacrificing the accuracy of the results: OCBSA-2021 provides the user with a computationally satisfactory band-straightening solution (10 s for a complete 10-lane membrane).

We expect our method to be of general value for DNA fragment and protein separation images, since geometric band distortion is a common problem for 2D gel electrophoresis, conventional gel electrophoresis, chemiluminescence IEF, and thin layer chromatography, etc. Moreover, our straightening algorithm can be applied to problems other than band straightening, such as aligning several profiles for comparison or aligning a profile with a reference gel for band matching.

Our method’s application field could be broadening to the straightening of other vertically shifted image structures, such as the correction of warped text in a scanned document prior to optical character recognition, or the registration of P and S images in seismic image analysis, etc.

Our research group is building an automatic tool for IEF image analysis and OCB detection. Our objective is to obtain a tool that provides a fluent user experience in terms of the analysis time and ease of use, is at least as accurate as the current visual analysis, and is free of inter-user variability and misinterpretation. Band straightening is a major IEF image enhancement that eases IEF image interpretation: it prevents faint, distorted bands from being ignored, and thus ensures reliable, sensitive OCB detection.

## Figures and Tables

**Table 1:** The average SD of the deformation introduced to the lanes without bands after processing with OCBSA-2016 or OCBSA-2021, for the training and test datasets.

	Band-Straightening Algorithm	Average SD (ΔY)
**Training—All** **(22 lanes)**	OCBSA-2016	1.214
	OCBSA-2021	0.976
**Test—All** **(18 lanes)**	OCBSA-2016	1.416
	OCBSA-2021	1.043

## Data Availability

The dataset analysed during the study are not publicly available and cannot be shared due to General Data Protection Regulation.
